# Medical teachers’ opinions about students with neurodevelopmental disorders and their management

**DOI:** 10.1186/s12909-020-02413-w

**Published:** 2021-01-06

**Authors:** Eloi Magnin, Ilham Ryff, Thierry Moulin

**Affiliations:** 1 Department of Neurology, Memory Center (CMRR), Centre Hospitalier Universitaire de Besancon, Besançon, France; 2 Clinical and Integrative Neuroscience, Research Laboratory 481, Bourgogne Franche-Comté University, Besançon, France

**Keywords:** Neurodevelopmental disorders, Mainstreaming (education), Staff development

## Abstract

**Background:**

Some students have neurodevelopmental disorders that might affect their academic and professional careers if they are not identified and addressed by specific pedagogic adaptations. The objective of this work was to describe medical teachers’ opinions of students with neurodevelopmental disorders and their management of these students.

**Methods:**

An anonymous cross-sectional electronic survey was performed to describe medical teachers’ opinions about the impact of neurodevelopmental disorders on the student’s life and on the medical teachers’ management. aThe survey was created, including visual analogic scales and free text, to assess teachers’ opinions from identification and assessment of neurodevelopemental burden on students and teachers, to their own knowledge about neurodevelopemental disorders and the specific pedagogic management available. The survey was sent to 175 medical teachers in 2019, of whom 67 responded. Quantitative descriptive statistics and qualitative analysis of free text were reported.

**Results:**

Many medical teachers report having encountered students who might have had neurodevelopmental disorders (dyspraxia 33%; dyslexia 46%; autism spectrum disorders 68%; attention deficit hyperactivity disorders 75%). Impact on students and on teachers was considered as important (mean VAS score for impact over 60/100 for all syndromes except for dyspraxia). Medical teachers’ self-reported knowledge about neurodevelopmental disorders (mean VAS score 43.9/100) and available pedagogical adaptations (mean VAS score 19.0/100) was limited. The teachers were concerned about ethical issues (mean VAS score 72.2/100) but were interested in receiving specialized training (mean VAS score 64.4/100).

**Conclusion:**

Medical teachers feel unprepared to manage students with neurodevelopmental disorders. They would be interested in specific training and procedures about the pedagogic management of these students.

**Supplementary Information:**

The online version contains supplementary material available at 10.1186/s12909-020-02413-w.

## Background

A common belief about medical students is that future physicians should have a “perfect mind” (i.e. be intelligent and sometimes “gifted”, empathetic, hardworking, and should present total psychological stability) [1, 2]. However, some medical students have neurodevelopmental disorders (NDDs) (e.g. 1.5 to 3% of medical students in the USA and UK have a Specific Learning Difficulty (SpLD), and this proportion is increasing over time (+ 13.4% from 2003 to 2007)) [3, 4]. NDDs, including Attention Deficit/Hyperactivity Disorders (ADHD), Autism Spectrum Disorders (ASD) [5], and SpLDs (such as developmental dyslexia, dysphasia, dyspraxia, and dyscalculia), induce specific cognitive and behavioral difficulties that might impact daily living activities including work and career [6]. About half of NDDs are not diagnosed in childhood [7]. Medical students with NDDs might therefore encounter specific difficulties during their academic careers. Moreover, those who were not diagnosed during childhood might be unaware of the origin of their difficulties and therefore also of the best adaptations and strategies to compensate. Additionally, they may lack pertinent information that would otherwise enable them to choose an adapted career pathway.

Very few articles have focused on the management of medical students with NDDs [3, 4], and those that do are frequently based on case reports and opinion papers. The lack of awareness among medical teachers about this subject and about adaptations for students with NDD is frequently discussed but has not been specifically assessed in the literature.

We hypothesized that due to the lack of available information, medical teachers would have little knowledge about NDDs and might underestimate this problem in the management of some of their struggling students. Our objective was therefore to describe medical teachers’ opinion of medical students with NDDs and their pedagogic management of these students.

## Methods

An anonymous, cross-sectional, descriptive survey was used to collect data about medical teachers’ opinions about students with NDD.

### The web-based survey (Additional file 1)

To our knowledge, no similar survey has been conducted in the literature. We therefore created a web-based survey focused on the pedagogic management of medical students with NDD, using the online tool SurveyMonkey [8]. An English translation of the survey is available in supplementary material #1.

Five medical teachers were invited to check the content validity of the survey (to reorder and rephrase items to ensure each question adequately captured the topic) [9]. Small changes were made to the order of the questions to improve the survey flow and sequencing of questions.

Visual Analog Scales (VAS) were chosen for the assessment of subjective characteristics or attitudes that cannot be directly measured because of a superior metrical characteristics compared to Likert scale [10]. In order to avoid inducing a bias in later questions in the survey, we did not use the term “NDD” in the title of the survey; we only referred to cognitive and behavioral disorders in medical students. For the same reason, we also did not provide diagnoses or specific symptomatologic terms in our examples.

The survey we created consisted of 26 items presented in 6 consecutive pages. Firstly, after asking the participants about their profession, four examples of medical students were briefly described, each with a paragraph showing the specific cognitive and behavioral difficulties they encountered during their medical studies (Table 1). The four cases corresponded to different kinds of NDD (case 1 = dyslexia, case 2 = ADHD, case 3 = ASD, case 4 = dyspraxia). Diagnoses and specific symptomatologic terms were not given to avoid inducing a bias in later responses. The difficulties reported in daily living activities were taken from a literature review [3, 4, 11–14] and from real examples of students with NDDs, in particular medical students, encountered in our adult neurodevelopmental center. This center was created in 2015 at Besançon University Hospital to improve screening, diagnosis and management of adults with NDD. The center has a multidisciplinary team including neurologists, psychiatrists, neuroscientists, neuropsychologists and speech therapists.

**Table 1:** Case descriptions

Case 1	The student’s written work (emails, reports, thesis, etc.) was of a very poor quality due to the spelling or grammar, to the extent that it made the text difficult to read and understand.
Case 2	The student has a great deal of difficulty organizing his/her work, a tendency to procrastinate, starts many tasks before finishing them, has difficulty prioritizing tasks and summarizing information, etc.
Case 3	The student has difficulty interacting with teachers, families, patients, other professionals, or other students (lack of empathy, irritability, use of inappropriately complex vocabulary, lack of self-reflection, inappropriate comments, etc.)
Case 4	Students who are particularly clumsy and have difficulty with technical actions that require dexterity (taking blood, suturing, etc.) despite having received what you would consider appropriate training.

After each example, participants were asked to use a four-point scale to estimate the frequency with which they had encountered this kind of medical student (never, rarely, sometimes, often). They were then asked to rate two VAS (0 = “no impact” to 100 = “severe impact”) focused on 1) the impact of these difficulties on the student’s academic career and future professional life, and 2) their own difficulties in teaching this kind of student. A free text space was then available for qualitative answers and observations.

After these four examples, respondents were then asked to use a VAS to indicate how aware they were that these students’ situations might have been induced by NDDs (0 = “not at all aware” to 100 = “very knowledgeable about this topic”).

Then, the participants were asked to estimate the frequency of NDDs in the general population in a multiple-choice question (1‰, 1, 5, 10, > 15%).

After this, we presented the participants with a one-paragraph overview of NDDs and their possible impact on medical students.

The participants were asked to use a VAS to evaluate their knowledge about possible pedagogic adaptations for students with NDDs (0 = “no knowledge about it” to 100 = “very knowledgeable about this topic”). A series of Yes/No items was used to evaluate their past use of these possible adaptations and a free text space was available for qualitative answers and observations to describe these adaptations.

We used two other VAS items to ask participants about 1) their interest in potential specific training about pedagogic management of students with NDDs (0 = “not at all interested” to 100 = “yes, very interested”) and 2) whether these issues might raise ethical problems for students, teachers, and future patients (0 = “not at all” to 100 = “yes, many”). A free text space was then available for qualitative answers and observations to describe these issues.

Finally, a free text space was available for qualitative answers and observations to describe the participants’ expectations of a training course focused on NDD.

### Study population

A web link to the electronic survey was sent to 175 medical teachers. These consisted of all the medical teachers at the University of Lyon who were participating in a pedagogic postgraduate degree exclusively for confirmed medical teachers applying for a grade advancement (*N* = 27), and all the medical teachers at the Besançon University medical faculty (*N* = 148). The link was sent by the faculty directorate to improve response rate. The number of invitations sent was based on a 30% estimated response rate, and the objective of a sample size of 50 responses, which would ensure the collection of enough data to allow meaningful interpretation [9].

Of the 175 medical teachers invited to participate, 67 responded to the survey, giving a response rate of 38%. Ten percent of the survey responses included missing data (not including free text responses).

### Analysis

#### Quantitative descriptive analysis

We exported the data from the SurveyMonkey database. We calculated the frequency for qualitative items, and mean, median, standard deviation, minimum and maximum for quantitative items.

Taking into account a confidence interval of 95% and the response rate of the survey, the margin of error was estimated at less than 10%. VAS responses lower than 30 and higher than 70 were considered significant and were added to the analysis of VAS results (i.e. > 70 = severe impact / good knowledge or interest / important ethical issues; < 30 = low impact / low knowledge or interest / few ethical issues).

As this is a descriptive study, we did not perform a statistical analysis.

#### Qualitative analysis

**Fig. 1:**
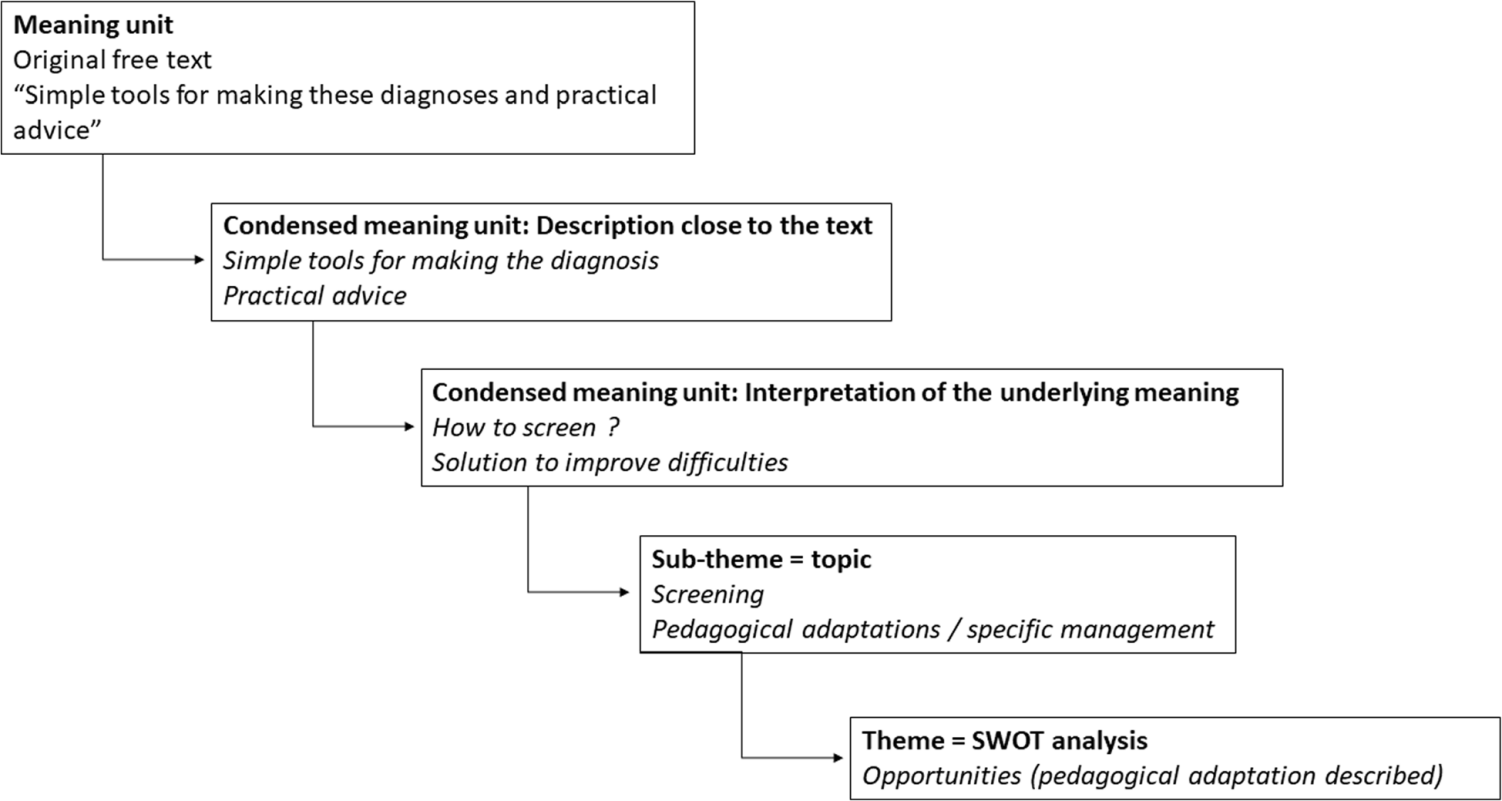
Examples of classification into meaning units, condensed meaning units, sub-themes and themes from content analysis of free text

We analyzed the free text responses by identifying each different item in the text. A specific workgroup made up of two medical teachers/neurologists and a neuropsychologist, all working in the regional NDD reference center, then reclassified the items into topics using qualitative content analysis [15]. This process is presented in Fig. 1. The frequency with which each topic was reported in our survey was collected. The topics were categorized into a pseudo-SWOT analysis (i.e. Strengths = positive perception of NDD; Weaknesses = negative perceptions of NDD; Opportunities = pedagogical adaptation described; Threats = ethical questions), which was later used to evaluate if it would be pertinent to highlight NDD in medical teachers’ teacher training [16]. We also built a colored wordcloud figure for each SWOT category, in which the font size corresponded to the frequency with which each topic was reported (for topics with frequency ≥ 5) [17].

## Results

### Quantitative descriptive analysis

Medical teachers reported having encountered our examples of students with possible NDDs quite frequently (i.e. the sum of reported frequency “sometimes” and “often”) except case four, which corresponded to dyspraxia (ASD > ADHD>Dyslexia> > Dyspraxia) (Table 2). The study participants generally considered that the NDDs would have a severe impact on the academic and professional careers of medical students, except dyspraxia (ASD > ADHD>dyslexia> > dyspraxia). They also reported that these disorders would have a high impact on medical teachers’ teaching methods (ASD > ADHD> > dyslexia>dyspraxia) (Table 2). The study participants underestimated the frequency of NDD in the general population, as the majority estimated that 5% or less had an NDD, whereas the epidemiological frequency in the general population is reported at at least 15% [6] (Table 3). Medical teachers’ perceptions of their knowledge of NDD (mean VAS for interest 43.9/100) and specific management (mean VAS for level of knowledge 19.0/100) was quite low. Interest in NDD training was quite high (mean VAS for interest 64.0/100). Ethical issues were frequently reported (mean VAS for interest 72.3/100) (Table 4).

**Table 2:** Medical teachers’ assessment of the impact of NDD on student life and teaching methods

		#1 (Dyslexia)	#2 (ADHD)	#3 (ASD)	#4 (Dyspraxia)
**Reported frequencies of encountering (%)**	Never	21	4	3	1
	Rarely	32	30	28	40
	Sometimes	45	46	54	29
	Often	2	20	15	4
	**Sometimes + Often**	**47**	**66**	**69**	**33**
**Impact on student** **(N = 67)**	Mean/Median VAS +/−SD (/100)	71.9 / 78 +/− 20.2	75.4 / 80 +/−17.4	83.4 / 89 +/− 16.6	61.2 / 60 +/− 21.9
	VAS Rating ≤ 30(little/no impact)	N = 4 (6%)	N = 1 (1.5%)	N = 1 (1.5%)	*N* = 4 (6%)
	VAS Rating ≥ 70(severe impact)	*N* = 46 (68%)	*N* = 50 (74%)	*N* = 55 (82%)	*N* = 23 (34%)
	[min-max] (/100)	[12–100]	[31–100]	[32–100]	[10–100]
**Impact on medical teacher** **(*N*** **= 67)**	Mean/Median VAS +/− SD)(/100)	60.1 / 62 +/− 21.5	70.8 / 72 +/− 18.8	74.0 / 80 +/−21	50.6 / 50 +/−19.9
	VAS Rating ≤ 30(little/no impact)	*N* = 8 (12%)	*N* = 3 (4.5%)	*N* = 4 (6%)	*N* = 12 (18%)
	VAS Rating ≥ 70(severe impact)	*N* = 28 (41%)	*N* = 41 (61%)	N = 46 (68%)	*N* = 10 (15%)
	[min-max]/100	[14–100]	[20–97]	[20–100]	[1–94]

**Table 3:** Estimated frequency of neurodevelopmental disorders in the general population

Estimated frequency of NDD	No. of responses (%) *N* = 61
1‰	0
1%	6 (10%)
5%	26 (43%)
10%	19 (31%)
> 15%	10 (16%)

**Table 4:** Medical teachers’ perception of their knowledge of and interest in NDD management

	Mean VAS score (/100)	Median VAS score (/100)	SD	[min-max]	VAS rating ≤ 30	VAS rating ≥ 70
Knowledge about possible NDDs in medical students with difficulties (N = 61)	43.9	50	+/− 30.7	[0–100]	*N* = 25 (41%)	*N* = 16 (26%)
Knowledge about pedagogic adaptations for students with NDDs (N = 60)	19.0	11.5	+/− 21.7	[0–90]	*N* = 47 (78%)	N = 2 (3%)
Interest in a specific pedagogic training about NDDs (*N* = 60)	64.0	67	+/− 26.9	[0–100]	*N* = 7 (11%)	*N* = 29 (48%)
Ethical issues in management of NDDs in medical students (N = 60)	72.3	76.5	+/− 21.3	[20–100]	N = 3 (5%)	*N* = 37 (61%)

In our study population, 16.3% of medical teachers reported having adapted their teaching methods for students with NDD.

### Qualitative analysis

**Fig. 2:**
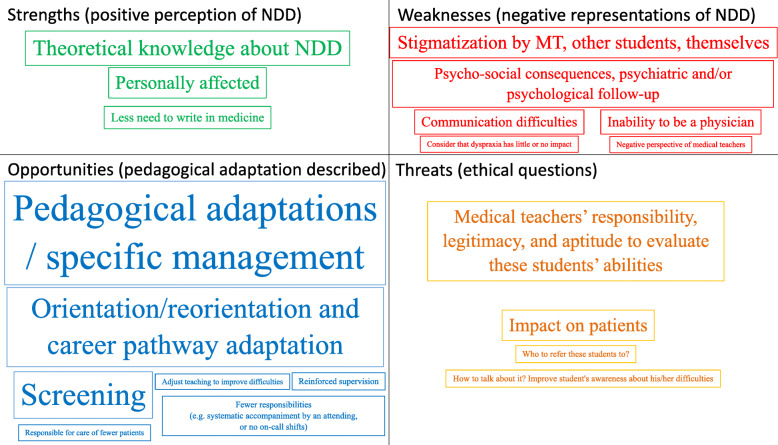
Pseudo-SWOT wordcloud showing perceptions and thematic topics related to NDD as reported by medical teachers (frequency ≥ 5). Green = Strengths (positive perception of NDD), Red = Weaknesses (negative representations of NDD), Blue = Opportunities (pedagogical adaptation described), and Orange = Threats (ethical questions)

Participants frequently completed the free text zones, and each zone usually addressed several topics. The number of participants who filled in each free text zone and the total number of items reported for each part of the survey can be found in Table 5. The total number of items reported was 254. These items were then reclassified into 48 different topics and 4 SWOT themes (i.e. Strengths = positive perception of NDD; Weaknesses = negative perceptions of NDD; Opportunities = pedagogical adaptation described; Threats = ethical questions), which are reported in Table 6 and Fig. 2.

**Table 5:** Number of free text responses and items reported for each part of the survey

Survey question	No. participants who responded in free text	No. items reported
#1 (dyslexia)	14	34
#2 (ADHD)	4	10
#3 (ASD)	5	6
#4 (dyspraxia)	10	16
Pedagogical adaptations	15	42
Ethical issues	28	93
NDD training expectations	26	53
Total		254

**Table 6:**
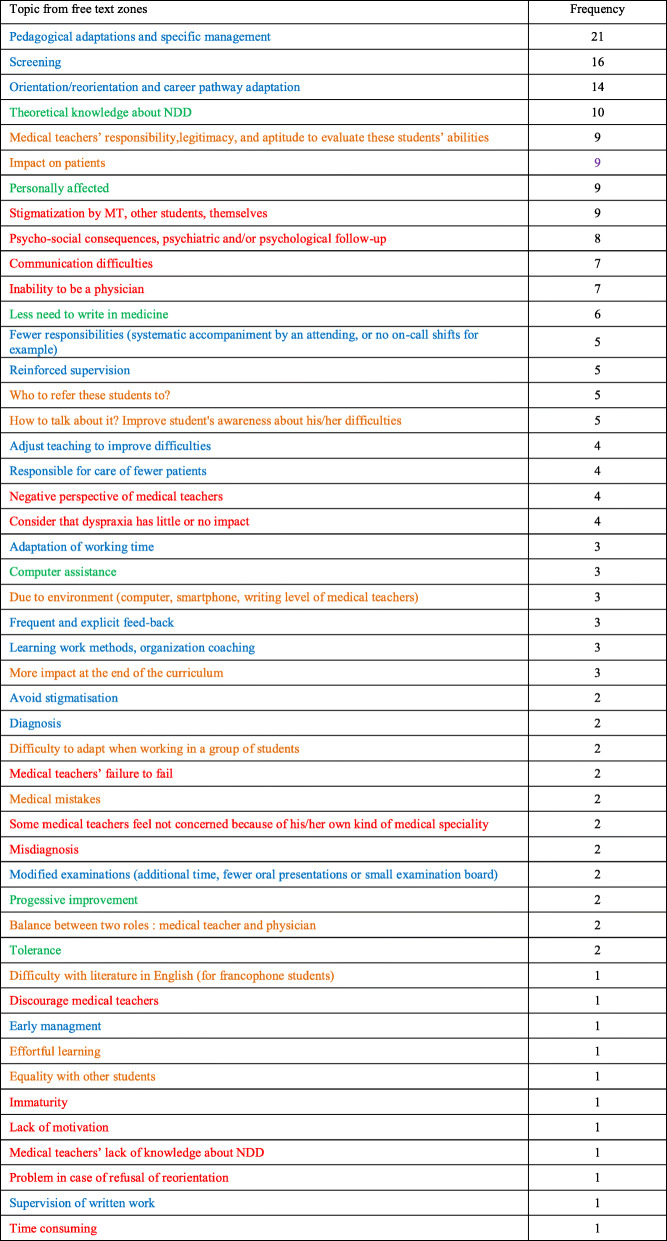
Report and frequency of thematic topics related to NDD extracted from free text zones. Green = Strengths (positive perception of NDD), Red = Weaknesses (negative perceptions of NDD), Blue = Opportunities (pedagogical adaptation described); and Orange = Threats (ethical questions)

Nine of the 67 medical teachers (13.7%) reported having an NDD themselves (4 with dyslexia, 3 with ADHD, and 2 unspecified NDD) and 6 (9%) reported that members of their family had an NDD (3 with ADHD, 1 with dyslexia, and 2 with unspecified NDD). These participants reported being more sensitive and forgiving towards students with NDD because they reported that they had encountered difficulties themselves in their academic and professional careers due to NDD.

## Discussion

To our knowledge, this is the first exploratory survey of the attitudes, knowledge and experience of medical teachers with regard to students with NDD. The response rate of 38% with 67 respondents was sufficient to interpret results even if this small sample size from only two universities might not be representative of the entire French population of medical teachers. A larger, nationwide confirmatory study would be interesting to replicate our results. A high rate of qualitative answers (items in free text zones) was a surprising result that might confirm the responders’ interest in this topic as reported by quantitative analysis (VAS).

Medical teachers reported encountering NDD students quite frequently. Respondents generally did not rate their awareness of the potential NDD origin of the examples very highly. Additionally, the frequency of NDDs in the general population was underestimated by our participants. These results support our hypothesis of a relative lack of information about NDDs. The respondents also reported that NDD symptoms would have a significant impact on both students and teachers. This profile of responses suggests that even if not accurately diagnosed, NDD conditions are probably frequently detected by medical teachers. It is therefore interesting to consider their role in screening for these disorders during the diagnosis of NDD in struggling students [18].

Moreover, medical teachers had low self-reported knowledge about pedagogical adaptations for struggling students as discussed in BEME guideline #56 [18], and only 16.3% of respondents reported having adapted their teaching methods for students with NDDs in the past. However, analysis of the free text showed that many medical teachers are aware of the potential adaptations and probably apply them in day-to-day practice even if the adaptations are frequently not formalized as an explicit pedagogical action.

Almost half of our population reported a high level of interest in receiving specific training on this topic. This interest was confirmed in the qualitative answers. These results highlight the need for specific training and procedures to help teachers to improve the management of medical students with NDD.

There was an overestimation of ASD frequency compared to other NDDs. Firstly, this may be because ASD is one of the most well-known NDDs, and is overrepresented by the media compared to other NDDs. Secondly, participants might have encountered students with other frequent psychiatric conditions that might have induced social cognition impairment (such as schizophrenia, bipolar disorders or personality disorders). Thirdly, ASD may be overrepresented among medical students, because some people with ASD are academically gifted.

The study participants considered that the symptoms described in example three (corresponding to autism spectrum disorder) would have the most impact on students and teachers. In the free text commentary, it was frequently reported that normal social cognition, especially empathy and communication skills, was almost mandatory for medical practice. The social interaction disorders presented in our example might also correspond to severe psychiatric disorders other than ASD, such as schizophrenia or other personality disorders. These disorders might induce more severe symptoms than ASD and lead to a total unsuitability for medical practice. It is possible that the participants postulated that the student had one of these severe psychiatric disorders rather than ASD and therefore over-estimated the impact of this condition on the student’s future professional activity. Moreover, communication and empathy skills can be improved by specific training and by clinical practice training for medical students [19], including in the ASD student population, who can benefit from explicit explanations and training in the relevant social mechanisms [11, 20]. The lack of knowledge about ASD might also induce stigma and prejudice [11]. Based on the responses given in items for example 4 of the survey and in the free text zone, our participants considered that dyspraxia would have a lower impact if the student’s career was oriented towards a medical specialty that did not require technical gestures, and it was considered as having little impact on medical teachers’ teaching methods. As dyspraxia is less common and therefore less well understood by medical teachers, its impact on daily living activities is probably underestimated. For example, dyspraxia induces severe difficulties in writing (speed and readability) that might affect academic performance during lectures and written examinations [14].

NDD syndromes (including ASD and dyspraxia) should therefore be well described in specific training about NDDs to improve knowledge about and personalized management of students with NDDs. The qualitative analysis showed that this training should also focus on screening NDDs, identification of practical support and points of contact who are experts in the pedagogical management of NDD, and practical teaching method adaptations and work adaptations to propose to the student during hospital placements (Table 7).

**Table 7:** Examples of important items to include in training on NDDs for medical teachers raised by the survey

What is an NDD? (epidemiology, different syndromes, impact on daily living)	
Suspicion of NDD in a student? (“red flags”; screening tools; contact with NDD reference center)	
Available pedagogical adaptations	
Advice about future orientation	
Procedure and points of contact with experts in the pedagogical management of NDD	
Ethical issues about NDD (stigmatization, teacher’s responsibility, impact on patients)	
Testimonies of students with NDD	

The topic of NDDs in medical students seems to raise many important ethical questions. Qualitative analysis of the free text zones showed that participants frequently reported issues about the role of medical teachers in NDDs and their diagnosis, and the potential conflict between medical teachers’ and physicians’ roles in the management of these students. The potential stigmatization of students with NDDs was also a major concern for the teachers. In addition, the potential risk to future patients treated by these students in case of misorientation and lack of adaptation was frequently highlighted. This “double-edged sword” effect associating benefit of the diagnosis and management and social burden at the same time has also been reported by students with ASD [11]. The problem of teachers’ “failure to fail underperforming trainees” reported in BEME Guideline #42 [21] was also reported by medical teachers, who did not feel they had the required competency to assess NDD students.

The SWOT categorization was chosen to evaluate if a specific focus on NDD was strategically adapted to a pedagogical approach. This analysis showed that several opportunities were available and/or partially set up to adapt the medical curriculum to students with NDD. However, it also highlighted several weaknesses due to negative perceptions of NDD by medical teachers. Additionally, threats included important ethical questions related to adapting medical education, and ultimately professional careers, for students with NDD, while keeping optimal treatment of patients as the primary goal.

## Conclusion

Medical teachers report having encountered many students who might have had NDDs, however their knowledge about NDDs and available pedagogical adaptations is limited. They also reported many potential ethical issues. Medical teachers would be interested in 1) specific training about the pedagogic management of students with NDD to improve their practice and 2) having designated expert medical teachers to give advice about teaching students with NDDs. No specific guidelines about the management of students with NDDs, such as a Best Evidence Medical Education (BEME) guideline, are currently available. Student-centered pedagogy [22] and well-being initiatives [23, 24] might take NND dimensions into account to produce a medical education program tailored to this specific population, which would benefit struggling students in particular [25].

## Supplementary Information

**Additional file 1.**

## Data Availability

The datasets used and/or analyzed during the current study are available from the corresponding author on reasonable request.

## Abbreviations

NDD: Neurodevelopmental disorders
SpLD: Specific Learning Difficulties
ADHD: Attention Deficit/Hyperactivity Disorders
ASD: Autism Spectrum Disorders
BEME: Best Evidence Medical Education
VAS: Visual Analogue Scales
SWOT: Strengths; Weaknesses; Opportunities; Threats
MT: Medical teacher
