# Supplementary material for: Medical teachers’ opinions about students with neurodevelopmental disorders and their management
Source: BMC Med Educ. 2021 Jan 6;21:16. doi: 10.1186/s12909-020-02413-w (PMC7789168; doi:10.1186/s12909-020-02413-w)
Supplement: Supplementary file 1 — Additional file 1. [file 12909_2020_2413_MOESM1_ESM.docx]

**Annexe 1 : Survey**


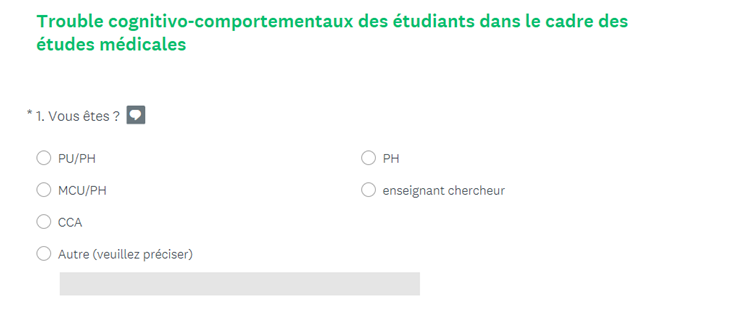


Cognitive and behavioral disorders in medical students

1. Please select your profession from the options below

- *Professeur* *Universitaire/Practicien hospitalier* (University professor-hospital attending)
- *Maitre des conférences universitaires/praticien hospitalier* (University lecturer-hospital attending)
- *Chef de clinique des universités-assistant des hôpitaux* (hospital fellow with teaching and research roles at the university)
- *Practicien hospitalier* (hospital attending)
- *Enseignant chercheur* (Teacher and researcher)
- Other (please specify)


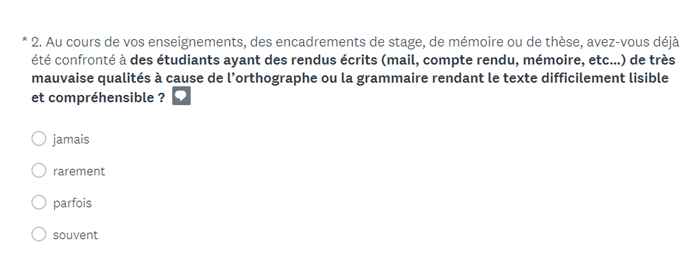


1. How often have you encountered a student corresponding to the following description? (In the context of teaching, mentoring, supervising a thesis). The student’s written work (emails, reports, thesis etc) was of a very poor quality due to the spelling or grammar, to the extent that it made the text difficult to read and understand.

- Never
- Rarely
- Sometimes
- Often


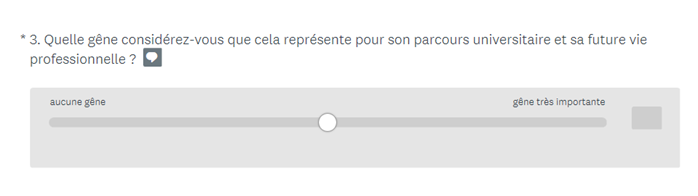


1. How severely would you rate the impact of this on the student’s academic career and future professional career (no impact — very severe impact)?


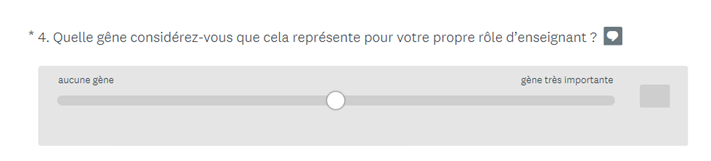


1. How severely would you rate the impact of this on your role as a teacher? (no impact — very severe impact).


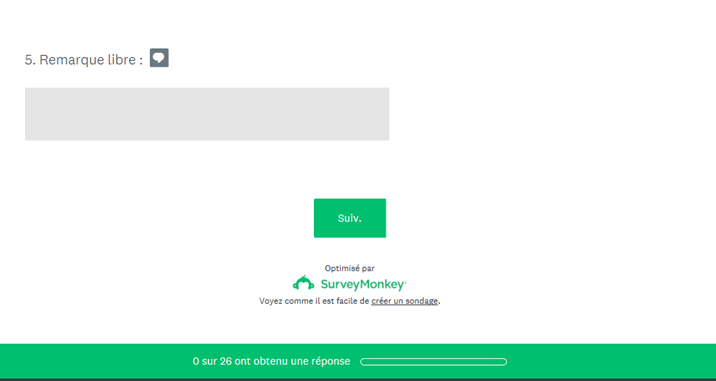


1. Free text


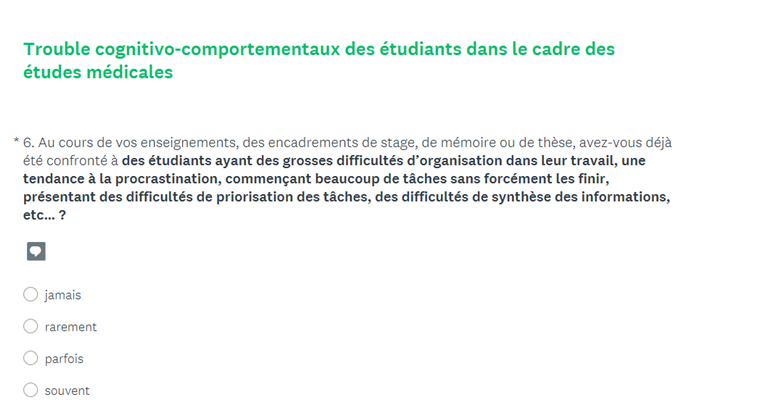


1. How often have you encountered a student corresponding to the following description? (In the context of teaching, mentoring, supervising a thesis). The student has a great deal of difficulty organizing his/her work, a tendency to procrastinate, starts many tasks before finishing them, has difficulty prioritising tasks and summarising information etc?

- Never
- Rarely
- Sometimes
- Often


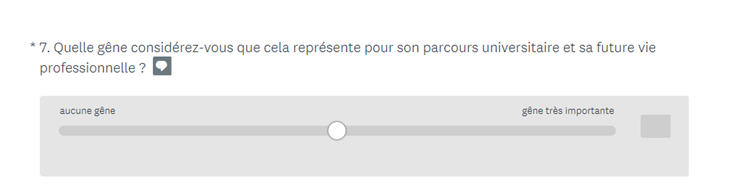


1. How severely would you rate the impact of this on the student’s academic career and future professional career (no impact — very severe impact)?


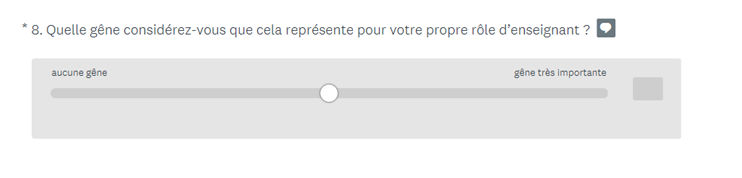


1. How severely would you rate the impact of this on your role as a teacher? (no impact — very severe impact).


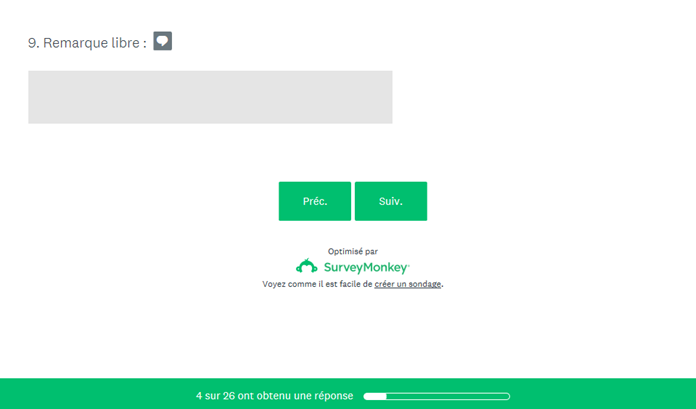


1. Free text


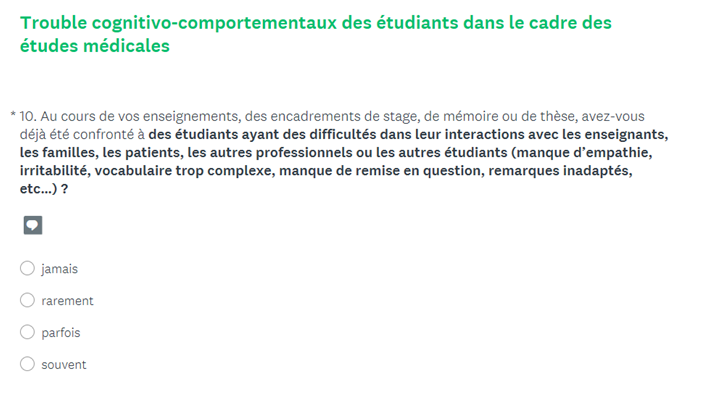


1. How often have you encountered a student corresponding to the following description? (In the context of teaching, mentoring, supervising a thesis). The student has difficulty interacting with teachers, families, patients, other professionals or other students (lack of empathy, irritability, use of inappropriately complex vocabulary, lack of self-reflection, inappropriate comments etc.)

- Never
- Rarely
- Sometimes
- Often


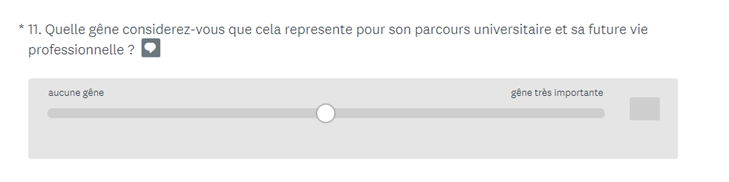


1. How severely would you rate the impact of this on the student’s academic career and future professional career (no impact — very severe impact)?


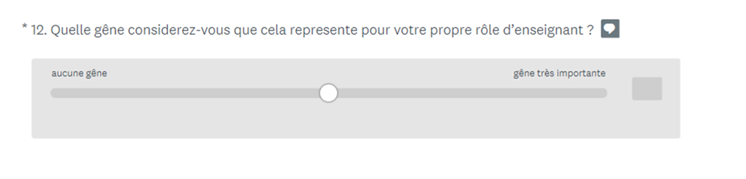


1. How severely would you rate the impact of this on your role as a teacher? (no impact — very severe impact).


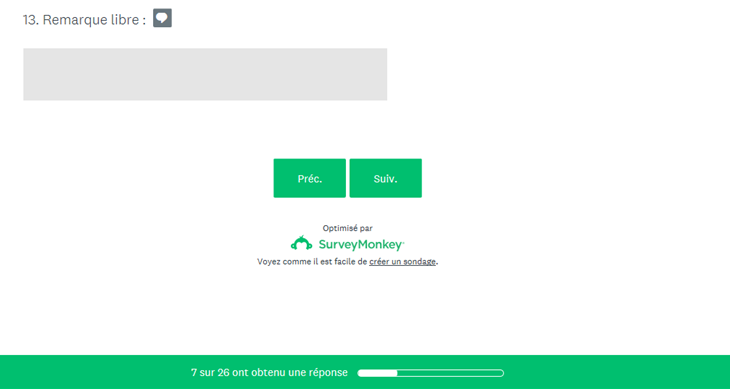


1. Free text


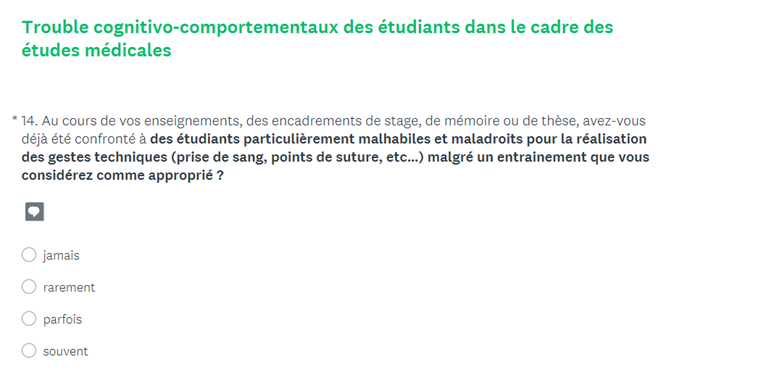


1. How often have you encountered a student corresponding to the following description? (In the context of teaching, mentoring, supervising a thesis). Students who are particularly clumsy and have difficulty with technical actions that require dexterity (taking blood, suturing etc) despite having received what you would consider appropriate training?

- Never
- Rarely
- Sometimes
- Never


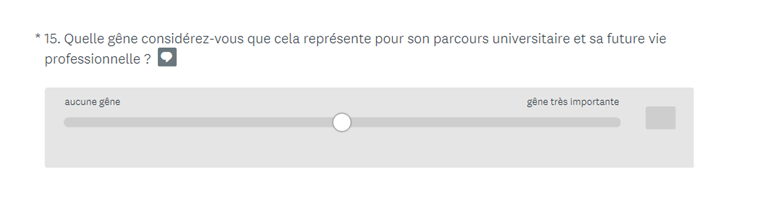


1. How severely would you rate the impact of this on the student’s academic career and future professional career (no impact — very severe impact)?


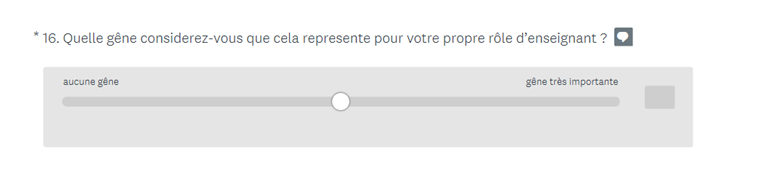


1. How severely would you rate the impact of this on your role as a teacher? (no impact — very severe impact).


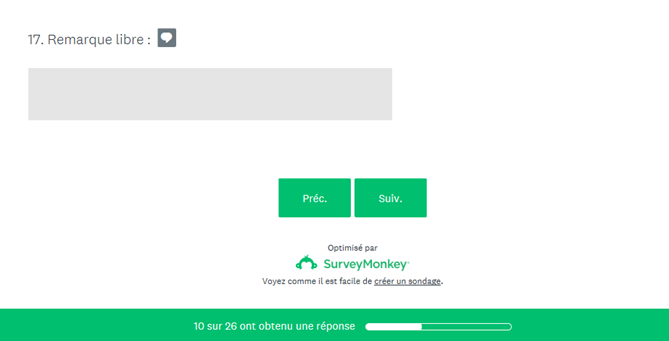


1. Free text


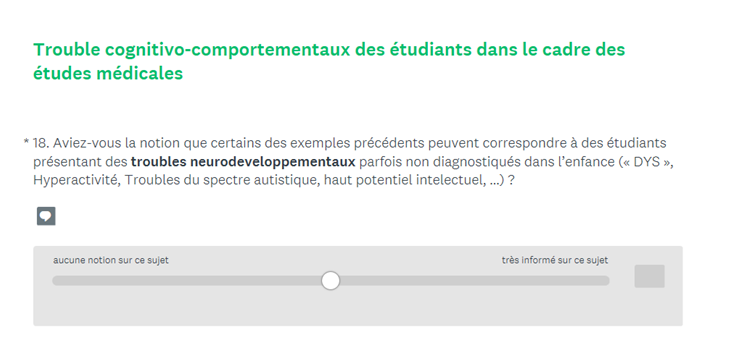


1. Are you aware that some of the examples given in the previous questions may correspond to students with neurodevelopmental disorders that may have remained undiagnosed through childhood (dys, hyperactivity, autism spectrum disorders, high intellectual potential…)? (Not at all aware – very knowledgeable about this subject)


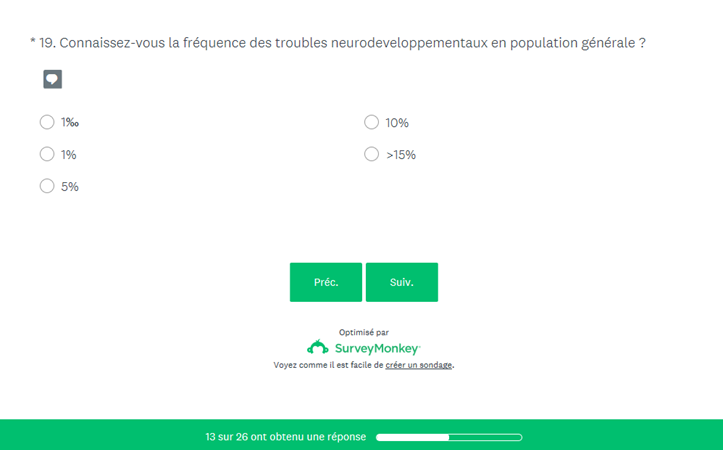


1.
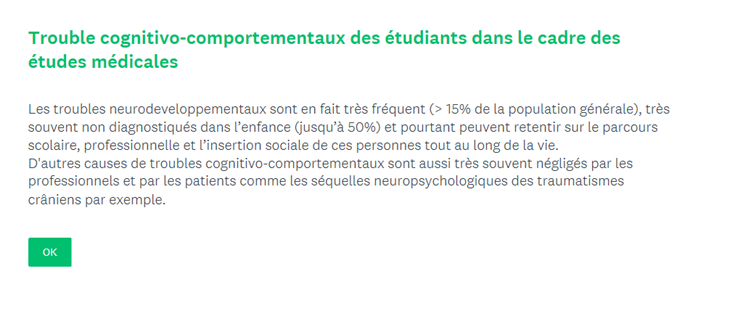
How would you estimate the frequency of neurodevelopmental disorders in the general population?

Cognitive and behavioural disorders in medical students

Neurodevelopmental disorders are in fact very common (˃15% of the general population), and are very often not diagnosed in childhood (up to 50%). They can, however, have a widespread impact throughout the person’s life, affecting their academic and professional careers and their social integration. Other causes of cognitive and behavioural disorders, such as neuropsychological sequellae from brain trauma, are also often neglected by professionals and patients.


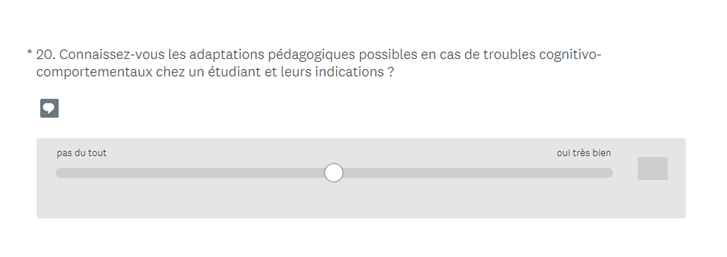


1. Do you know about possible adaptations to teaching methods and their indications for students with cognitive or behavioural disorders? (no knowledge about it — very knowledgeable about it)


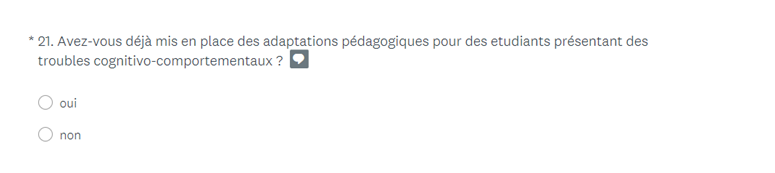


1. Have you ever adapted your teaching methods for students with cognitive or behavioural disorders? (Yes or no)


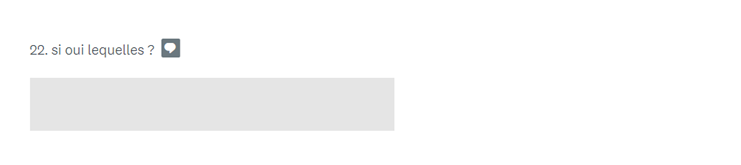


1. If yes, how?


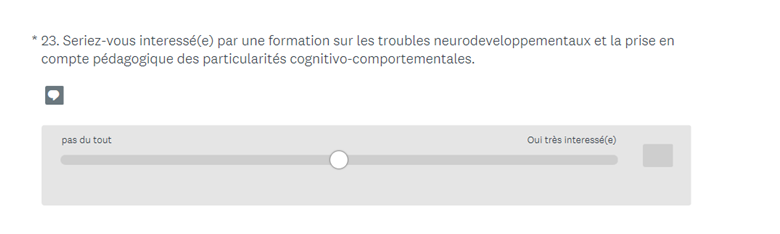


1. Would you be interested in attending training on neurodevelopmental disorders and pedagogic management of the specificities of cognitive and behavioural disorders? (Not at all interested — yes, very interested)


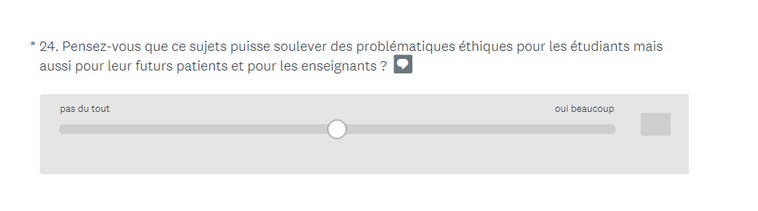


1. Do you think that these issues could raise ethical problems for teachers, students and their future patients? (Not at all — yes, many)


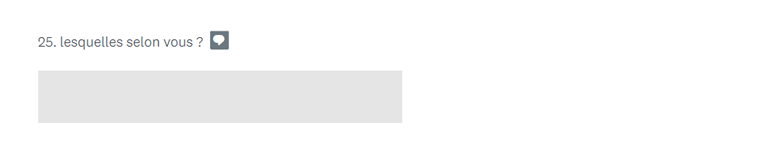


1. If yes, please specify.


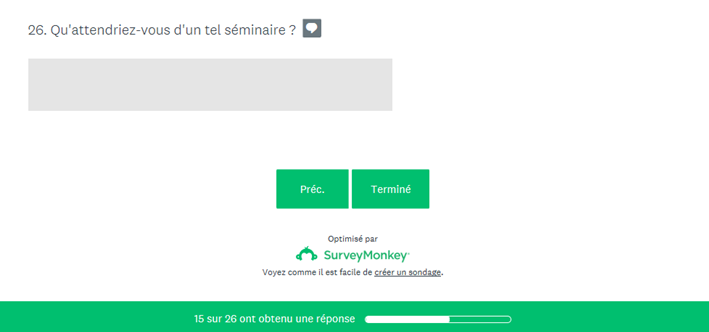


1. What would you expect from a training session on this subject?
